# Strengthening community health supply chain performance through an integrated approach: Using mHealth technology and multilevel teams in Malawi

**DOI:** 10.7189/jogh.04.020406

**Published:** 2014-12

**Authors:** Mildred Shieshia, Megan Noel, Sarah Andersson, Barbara Felling, Soumya Alva, Smisha Agarwal, Amnesty Lefevre, Amos Misomali, Boniface Chimphanga, Humphreys Nsona, Yasmin Chandani

**Affiliations:** 1 JSI Research & Training Institute, Inc., Nairobi, Kenya; 2 JSI Research & Training Institute, Inc., Arlington, VA, USA; 3 Johns Hopkins University, Baltimore, MD, USA; 4 JSI Research & Training Institute, Inc., Lilongwe, Malawi; 5 Ministry of Health, Blantyre, Malawi

## Abstract

**Background:**

In 2010, 7.6 million children under five died globally – largely due to preventable diseases. Majority of these deaths occurred in sub–Saharan Africa. As a strategy to reduce child mortality, the Government of Malawi, in 2008, initiated integrated community case management allowing health surveillance assistants (HSAs) to treat sick children in communities. Malawi however, faces health infrastructure challenges, including weak supply chain systems leading to low product availability. A baseline assessment conducted in 2010 identified data visibility, transport and motivation of HSAs as challenges to continuous product availability. The project designed a mHealth tool as part of two interventions to address these challenges.

**Methods:**

A mobile health (mHealth) technology – cStock, for reporting on community stock data – was designed and implemented as an integral component of Enhanced Management (EM) and Efficient Product Transport (EPT) interventions. We developed a feasibility and acceptability framework to evaluate the effectiveness and predict the likelihood of scalability and ownership of the interventions. Mixed methods were used to conduct baseline and follow up assessments in May 2010 and February 2013, respectively. Routine monitoring data on community stock level reports, from cStock, were used to analyze supply chain performance over 18–month period in the intervention groups.

**Results:**

Mean stock reporting rate by HSAs was 94% in EM group (n = 393) and 79% in EPT group (n = 253); mean reporting completeness was 85% and 65%, respectively. Lead time for HSA drug resupply over the 18–month period was, on average, 12.8 days in EM and 26.4 days in EPT, and mean stock out rate for 6 tracer products was significantly lower in EM compared to EPT group.

**Conclusions:**

Results demonstrate that cStock was feasible and acceptable to test users in Malawi, and that based on comparison with the EPT group, the team component of the EM group was an essential pairing with cStock to achieve the best possible supply chain performance and supply reliability. Establishing multi–level teams serves to connect HSAs with decision makers at higher levels of the health system, align objectives, clarify roles and promote trust and collaboration, thereby promoting country ownership and scalability of a cStock–like system.

In 2010, 7.6 million children under five died globally – largely due to preventable diseases including pneumonia (14%), diarrhea (10%), and malaria (7%) [1]. Ninety–nine percent of these deaths occurred in low–resource settings and nearly half (47%) in Sub–Saharan Africa in communities where people have limited or no access to life saving interventions and medical supplies. Integrated Community Case Management (iCCM) is a strategy designed to bring care and treatment for childhood pneumonia, diarrhea, and malaria closer home by training community health workers (CHW) in the identification and treatment of common childhood illnesses [2,3]. iCCM involves training CHWs on essential health packages and ensuring that they have the medicines needed to manage and treat illnesses among children under five years of age. Continuous access to these medicines by CHWs requires a well–functioning supply chain across all levels of the health care system. However, the supply chain systems of many resource–constrained countries function poorly and face a myriad of challenges [4], including but not limited to shortage of human resources, weak inventory management, low supply chain skills among health workers, and a lack of data visibility and utilization for sound decision making.

In 2008, the Government of Malawi (GoM) initiated iCCM as a strategy to reduce child mortality. The program entailed training an existing cadre of CHWs, known as Health Surveillance Assistants (HSAs) to treat children in the community. HSAs are posted nationwide to serve communities at a ratio of 1:1000 population. By September 2011, 3296 HSAs had been trained in integrated management of childhood illness (IMCI) [5]. With the implementation of the iCCM strategy in Malawi, mortality among children under five years decreased from 225 deaths per 1000 live births in 1990 to 71 per 1000 live births in 2012 [6], and the country is considered on track to achieve Millennium Development Goal (MDG) 4, to reduce child–mortality by two–thirds, by 2015.

Despite the gains made towards MDG 4, the health care infrastructure in Malawi still faces challenges, among them weak supply chain systems. This affects continuous health product availability at the community level, consequently undermining the full effectiveness of the iCCM strategy. In order to identify the constraints associated with maintaining regular product availability, Supply Chains for Community Case Management (SC4CCM) in collaboration with the Ministry of Health (MOH), conducted a formative assessment across 10 districts in Malawi (Nkhatabay, Nkhotakota, Mulanje, Kasungu, Nsanje, Machinga, Mzimba North, Zomba, Nchitsi, and Salima) in 2010 [7]. The assessment identified poor availability and limited use of logistics data (ie, low data visibility) across all levels of the health system, low motivation among HSAs and transport challenges such as difficult terrain and long travel time for HSAs to collect products as key barriers to continuous product availability. This assessment identified additional opportunities of using mobile phones to promote data visibility as 89% of the HSAs surveyed had mobile phones, 80% of HSAs and health facility (HF) staff had continuous network coverage at their place of work and all districts surveyed had computers and access to the Internet. The survey also found high levels (80%) of bicycle ownership among the HSAs [7].

To address the identified constraints related to data visibility, motivation and transport, SC4CCM designed and piloted cStock, a mHealth tool for community–level reporting of stock on hand data and resupply of 19 health products managed by HSAs. cStock was nested within two broader interventions, namely, Enhanced Management (EM) and Efficient Product Transport (EPT), to address challenges in motivation of HSAs and transport to the health facilities, respectively. The primary objective of this paper is to assess the feasibility, acceptability, and effectiveness of cStock as a mHealth strategy for improving data visibility and reducing stockouts of health products used at the community level. Additionally, the study will explore the added effects of the team approach deployed through EM in improving supply chain performance.

## Program description

cStock is an SMS and web–based reporting and resupply system that is used by HSAs to report stock data via SMS through their personal mobile phones. cStock calculates HSA resupply quantities and sends this information to HF staff to use to pick and pack products for HSAs and notify them about a collection time.

cStock was designed using a consultative, user–centered and iterative process. Potential users at all levels of the health system provided inputs based on their experience with the existing manual reporting and resupply system. Information on the existing flow of data across levels of the health system was combined with inputs from supply chain specialists to ensure the system was based on supply chain best practices. In designing the workflows and dashboard for cStock, an important criterion was to ensure the health care workers and managers at each level would have access to data most relevant to them, at the right time and in a format that could be easily accessed, interpreted and used for decision making. HSAs and HF staff would interact with cStock using SMS messages on their own phones, while district and central level staff would receive alert messages from cStock on their own phones. District and central level staff would use computers to access the web–based dashboard for reports. The dashboard was redesigned six months after implementation so that district and central level users could incorporate their experience interacting with the system into the redesign and prioritize metrics and visuals most useful for their day–to–day operations.

cStock is a key component of both the EM and EPT intervention packages. The EM intervention addresses challenges related to data availability and visibility, as well as low motivation among HSAs while the EPT intervention addresses challenges of transport in addition to data visibility. The additional component of the EM intervention was District Product Availability Teams (DPATs). These are multilevel quality improvement teams that use data supplied by cStock to monitor performance of the supply chain and make informed supply chain decisions. In contrast, the additional component of the EPT intervention consisted of training all HSAs on bicycle maintenance, provision of a basic tool kit, and the use of a continuous review inventory control system.

HSAs and HF staff in six districts where the project was piloted were trained on the use of cStock for reporting and resupply and used their own phones to register with cStock. Training was conducted using a cascade approach. The project trained a group of trainers consisting of central and district level staff from pilot districts as TOTs and they in turn trained the HSAs, HF and district staff. Both intervention groups were trained over a 2–day period, with one day dedicated to training users on how to register and use cStock. HSAs in the EM group were trained to send reports to cStock using a fixed monthly reporting schedule between the last day of the month and second day of the next month, while those in the EPT group were trained to send reports to cStock at any time during the month when they planned to travel to the HF giving them the flexibility to report and receive product. Once trained, HSAs were provided with job aids to carry back to their facilities and expected to start using cStock immediately. Implementation support for cStock was provided through group messages sent by the system administrator to users to correct common errors, automated messages sent directly by the system to users in response to formatting errors, and field visits made by Ministry and project staff to monitor and reinforce good practices.

**Figure 1:**
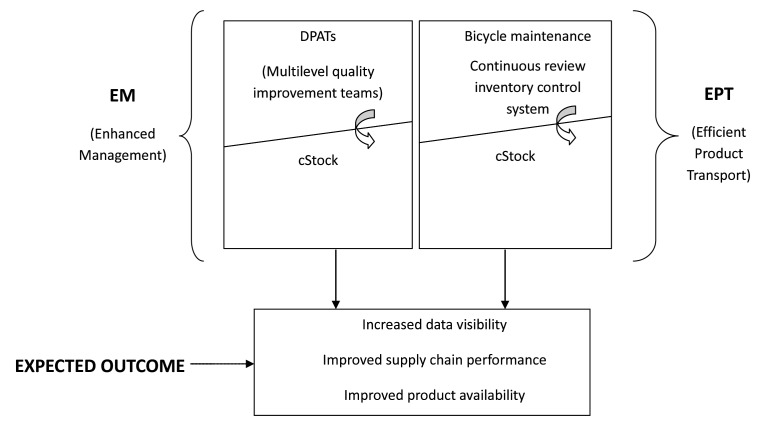
Components of the two intervention groups (EM – Enhanced Management and EPT – Efficient Product Transport). DPAT – district product availability teams.

In designing the interventions, the project identified feasibility and acceptability of cStock as critical components to enhancing uptake and laying the foundation for country ownership and scalability. See **Figure 1** for a visual representation of the intervention design.

## METHODS

### Study site

Malawi is a landlocked country that covers an area of 118 484 km^2^ and shares boundaries with Zambia in the west, Mozambique in the east, south and southwest, and Tanzania in the north. The country is divided into three administrative regions, namely the northern, central and southern regions with 28 districts in total [8]. The study purposefully selected 10 out of Malawi’s 28 districts for the 2010 baseline assessment, in consultation with stakeholders. Selection criteria for the districts included the existence of a functioning iCCM program, a balance of iCCM partner support, and a relatively balanced geographical coverage across the three administrative regions of the country.

### Evaluation framework

**Figure 2:**
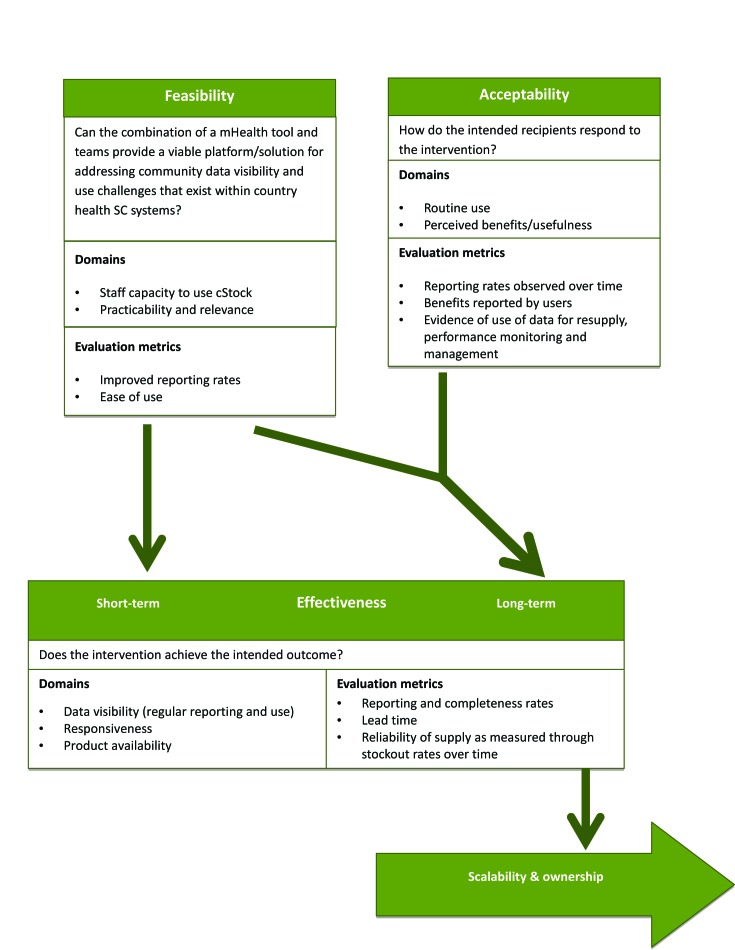
Feasibility, acceptability and effectiveness framework.

To evaluate the effectiveness of the interventions, the project designed a feasibility and acceptability framework (**Figure 2**) that identifies key domain areas as important measures for predicting the likelihood of long–term effectiveness, scalability and ownership of an intervention.

In this framework a **feasible intervention** is one that seeks to address a defined problem and that provides a viable solution or platform for solving the problems within existing health system structures and staff capacities in a country. The solution should be easy to learn, quick and easy to use, with few or no difficulties experienced by the user. For this evaluation the measures of feasibility are:

• Improved reporting rates, and

• HSAs and HFs have the necessary skills and ability to use cStock.

An **acceptable intervention** is one that users perceive as valid, reliable, and beneficial; users are satisfied that the intervention meets their needs by helping them adequately solve a problem or make improvements in their daily work. Acceptability is achieved when the majority of users use the intervention on a regular basis for supporting routine tasks. For this evaluation the measures of acceptability are:

• cStock has become the primary means for HSAs to order or request health products from their resupply point,

• cStock has become the primary tool for HF staff to use for resupplying HSAs, and

• Teams use data from cStock to measure, monitor, and improve supply chain performance.

An **effective intervention** is one that achieves the intended or desired outcome. In this study the desired outcomes across the two intervention groups are improvements in data visibility, responsiveness and supply reliability. For this evaluation effectiveness is measured and compared across the EM and EPT groups as:

• Improved data visibility measured as improved reporting rates and complete reporting rates,

• Improved responsiveness measured as improved lead times, and

• Improved supply reliability measured as reductions in stock out rates.

### Assessment tools and study groups

The project employed mixed methods to conduct baseline and follow up assessments in May 2010 and February 2013, respectively. Permissions for the assessments were obtained from the MOH. The quantitative survey tool was adapted from the Logistics Indicator Assessment Tool (LIAT) originally developed by the USAID | DELIVER PROJECT [9], including questionnaires, inventory assessment forms, and key informant interview guides. The survey was tailored to the district, HF and community level, to capture processes, behaviors, and product availability data along each step in the supply chain and to measure indicators of intervention implementation.

A local evaluation partner, the Malaria Alert Center (MAC), was selected through a competitive process to lead data collection activities. For quantitative data collection, enumerators were trained to interview HSAs and other staff managing supplies of medicines. Data collectors used Nokia e71 and e63 smart phones loaded with Data Dyne’s Magpi application, which allowed for streamlined data entry and immediate review of data after uploading records to a web–based system. Data were saved on a daily basis to external memory cards to prevent loss. Frequencies were carried out using STATA version 11.

In addition to the cross–sectional data collection at baseline and follow up, focus group discussions (FGDs) were carried out in February 2013 (at follow up) by a team of two researchers from MAC, with HSAs and health facility staff in all six pilot districts. Two FGDs per district, with 6–10 participants each, were held with staff from several health facilities. In each district, one FGD included only HSAs (one male and one female per HF), and the other included only HF staff handling CCM products (HSA Supervisors, Drug Store In–Charge / HF In–Charge). A thematic guide was used to collect qualitative information around community supply chain data visibility and use, with particular focus on contributions made by the cStock system. For the EM group, FGDs also gathered views on the EM intervention and achievements made by the DPATs, as well as lessons learned about the intervention. The data collection team transcribed notes immediately after discussions, and qualitative results were thematically synthesized using a notes–based analysis.

Routine monitoring data on HSA drug stock level reports, submitted using cStock, were utilized to study supply chain (SC) performance trends over time between the EM and EPT groups. These data were retrieved for the 18–month period from January 2012 to June 2013. The web–based cStock dashboard provided reports showing monthly stock reporting rates, average time taken between sending an order request and receiving health products (lead time), product availability, and stock–outs for these time periods for the six intervention districts (three for EM and three for EPT). The average values of these key indicators were calculated for the 18–month period and paired Student’s t–tests were conducted to compare the trends between the EM and EPT groups. **Table 1** presents an overview of the various data sources and frequency of collection.

**Table 1:** Malawi data sources

Data source/tool	Description and methods	Frequency of collection	Sample size
Logistics Indicators Assessment Tool (LIAT) used to measure feasibility and acceptability parameters	Quantitative data collection to determine supply chain (SC) performance at HSA and higher levels; longitudinal comparisons of key indicators using Facility–based survey, for assessment of stock levels, reports, and storage conditions	Baseline Midline	- Districts: n = 6 - HFs: n = 51 (BL);n = 48(ML) - HSAs: n = 100 (BL); n = 159 (ML) *Includes EM & EPT groups
FGDs used to measure the feasibility and acceptability parameters	Qualitative data collection to learn about community and HF user perceptions of cStock and EM by conducting FGDs with HF staff and HSAs in the 3 districts using cStock and DPAT (EM group)	Midline	Six (6) FGDs total, 2 per district: one (1) with HSAs (1 male and 1 female per HF), and the other (1) with HF staff who handle CCM products (ie, HSA Supervisors, Drug Store In–Charge / HF In–Charge). Each FGD had 6–10 participants, from 3–4 HFs per district. *Includes EM group
cStock used to measure the effectiveness parameters	Routine SMS reporting system for all products managed by HSAs. Web–based dashboard reports for SC performance monitoring of trends over time providing monthly data accessible by time and/or district–based query reports from the cStock dashboard Reports include: - Reporting rates - Stock status - Lead times - Order fill rates	Jan 2012–June 2013	Registered cStock users as of June 2014: - Districts: n = 27 (including 6 EM & EPT) - HFs: n = 522 - HSAs: n = 2707 (including n = 646 from EM & EPT districts) *Includes EM & EPT groups

LIAT – logistics indicators assessment tool, SC – supply chain; HF – health facility, HSA – health surveillance assistant, SMS – short message service, FGD – focus group discussion, EM – enhanced management, EPT – efficient product transport, BL – baseline, ML – midline

HSAs were the primary unit of survey analysis. This paper presents two sets of samples, one from the baseline and follow up surveys, the second from backend data collected via cStock where the sample is comprised of all HSAs who ever registered on cStock at any point in the program cycle. 56 HSAs from the EM districts, and 44 HSAs from the EPT districts were sampled at baseline, 81 HSAs from EM districts and 78 HSAs from the EPT districts were sampled at follow up. The cStock sample of HSAs registered in each group as of June 2014, is n = 393 HSAs in EM, and n = 253 HSAs in EPT (total n = 646).

### Sampling strategy

After the formative assessment, the project formed three groups of three, three, and four districts from the ten districts by matching geographical and demographic characteristics, and other external dimensions including iCCM partner coverage, prevalence of diarrhea, malaria, and cough, as well as baseline HSA iCCM product availability, to create comparable groups. The three groups were randomly assigned to receive the EPT intervention (three districts), the EM intervention (three districts) and no intervention (four districts). This paper presents results from the six intervention districts where the project implemented EM and EPT, and thus where cStock was tested.

As the project chose 10 districts purposefully for evaluation, a representative sample of HFs was chosen from within the 10 districts at baseline and follow up. Probability proportional to size sampling was used to randomly select health centers based on number of associated village clinics (sites where HSAs work), and approximately three HSAs who manage health products were randomly selected per health center and visited at their village clinic.

## RESULTS

Study findings on the feasibility and acceptability of cStock, as well as acceptability of EM, are presented below from the follow up LIAT survey and FGD findings (**Tables 2**** and ****3**).

**Table 2:** Feasibility of cStock – LIAT Findings (Midline only)*

Domain	EM, No. (%)	EPT, No. (%)
**Staff capacity to use cStock**		
District staff comfort level navigating cStock dashboard:		
– Very comfortable	3 (67)	3 (33)
– Comfortable	3 (33)	3 (33)
– Somewhat comfortable	3 (0)	3 (33)
District staff receive SMS alerts on low stocks from cStock	3 (100)	3 (100)
HSA supervisor reports they are able to send order–ready message and receive cStock messages for HSAs	25 (96)	23 (87)
**Practicability**		
HSAs are able to prepare and send report to cStock within an hour	81 (86)	77 (91)
**Challenges associated with use**		
HSAs reporting challenges transmitting the cStock reports by SMS†	81 (20)	77 (29)
Drug Store In–Charge encountered challenges using cStock‡	24 (33)	22 (23)
HSA Supervisor encountered challenges using cStock§	25 (8)	23 (9)

LIAT – logistics indicators assessment tool, HSA – health surveillance assistant, SMS – short message service, EM – enhanced management, EPT – efficient product transport

*Results presented after 18 months of follow–up.

†HSA challenges include network not always available (n = 16), getting error messages (n = 8), repeated nags (n = 2), no place to recharge (n = 1).

‡Drug Store In–Charge challenges include insufficient or no training (n = 5), quantities do not make sense (n = 2), phone issues (n = 2), HSAs not sending reports through cStock/abnormal SOH provided by some HSAs (n = 2), ready message not received (n = 1), network (n = 1), understock (n = 1).

§HSA Supervisor challenges included quantities do not make sense (n = 2), getting error messages (n = 1), no place to charge battery (n = 1).

**Table 3:** Acceptability of cStock and EM—LIAT Findings (Midline only)*

Domain	EM, NO. (%)	EPT, No. (%)
**Routine use**		
**cStock:**		
HSAs reported using cStock to place emergency orders.	81 (94)	78 (88)
HSAs reported using cStock to send receipts after collecting products.	81 (100)	77 (99)
District staff reported monitoring stock levels and contacting HF when low stock.	3 (33)	3 (0)
District staff use cStock to target HFs for supervision	2 (67)	3 (0)
**DPAT:**		
HF staff reported monitoring the performance of their DPAT using EM	56% consult cStock; 40% consult Resupply Work sheet (25); 4% do not use anything	NA
**Perceived benefits**		
**cStock:**		
HSAs use cStock as the primary means for ordering health products from their resupply point.	81 (97)	78 (91)
HF Drug Stores use cStock to determine quantities to resupply HSAs	25 (92)	23 (91)
**DPAT:**		
HSAs who attended DPAT meetings reported that a variety of SC topics were covered, including:		
– Reporting, including timeliness and completeness	58 (79)	NA
– Stock management, including expiries and product availability	58 (53)	NA
– Performance improvement	58 (33)	NA
– Performance and recognition plans	58 (13)	NA
HSAs reporting that their DPAT has performance targets to help improve the way they manage products	81 (85)	NA

LIAT – logistics indicators assessment tool, SC – supply chain; HF – health facility, HSA – health surveillance assistant, EM – enhanced management, EPT – efficient product transport, DPAT – district product availability team, NA – not applicable

*Results presented after 18 months of follow–up.

Because outcomes related to effectiveness are determined in the model by the aspects of feasibility and acceptability, we first present the findings on feasibility and acceptability.

### Feasibility of cStock for users

The feasibility of cStock was evaluated by looking at **staff capacity to use cStock, practicability, and relevance**. Follow up findings (February 2013) demonstrated an improvement in reporting of community–level stock data using the cStock system, with 85% of HSAs in six districts sending reports to cStock. In comparison, community data was much less visible at baseline, with only 61% of HF staff across six districts (n = 51) reporting HSA supply chain data up to district level, and only 29% of those HFs reporting this data disaggregated from HF data. **Table 2** presents a summary of feasibility findings from the follow up survey.

A majority of users at all levels of the system found the cStock technology **easy to learn**, with 80% of HSAs and 92% of HF staff in the EM group, and 71% of HSAs and 91% of HF staff in EPT group able to send and receive messages without any challenges. HSAs also noted that cStock is **easy to use**, with the majority able to “type in the SMS” without any challenges. District staff in all three EM districts and two of three in EPT districts reported feeling comfortable accessing and navigating the dashboard. Minor use challenges reported by HSAs and HF staff were mainly infrastructural in nature, related to poor network penetration and lack of charger or a place to charge phones.

FGD findings highlight the **practicability** of cStock as shown by the convenience of the system in enabling HSAs to send data on stock levels at any time, and corroborate the survey findings where majority (86%) of HSAs were able to compile and send stock data within an hour. One HSA from the EM group explains: “… *[cStock] is also good because we are able to send the messages at any time even in the night and we get the response in time*.”

FGD findings related to the theme of **relevance to users** include HSAs description of cStock and its components as a fast system for sending messages and receiving feedback. An HSA Supervisor in the EPT group offered: “*Yes, [cStock is] very useful because communication is very fast between the HSA and the In–Charge, whenever they want emergency order they receive feedback right away from the health facility.*”

HSAs and HF staff also perceived cStock as a linkage that facilitates communication between levels from the villages to the HSAs, HSAs to the HF. One HSA Supervisor in the EPT group offered, “*… [cStock] is a linkage between the HSAs and the supervisors, even the In–Charge because when the products are ready we just send the message to let them know that the drugs are ready. While before it was hard to reach every HSA*.” Users also noted that the cStock system helps users remember to take necessary actions, for example one HSA in the EPT group stated, “*cStock is good because it reminds you what to do, like if you have one day left to send the report it always reminds you that you have one day left.”*

### Acceptability of cStock

The acceptability of cStock was evaluated by looking at its level of **routine use**, **its effect on users’ daily work,** and **perceived benefits** identified by the user. Data for the six intervention districts, analyzed over 18 months from Jan 2102 – June 2013, showed a steady increase (79% to 99% in EM and 71% to 90% in EPT) in routine use of cStock to report on stock levels averaging at 94% for EM, 79% for EPT. Follow up findings also showed that a large majority (97% in EM and 91% in EPT) of HSAs reported that cStock had become their primary means for ordering or requesting health products from their resupply point. Additionally, 92% of health facility Drug Store In–Charges in EM and 91% in EPT reported using cStock to determine the quantities to resupply to HSAs. **Table 3** presents a summary of acceptability findings from the follow up survey.

In addition to routine orders, HSAs also reported using cStock as the primary means for submitting emergency orders. While at baseline, 52% of HSAs across six districts (n = 98) reported travelling to the HFs to submit emergency orders, this percentage dropped to 28% in EM and 21% in EPT at follow up, with the majority (94% in EM, 88% in EPT) of HSAs now using cStock to send emergency orders. All HSAs (100% in EM, 99% in EPT) also reported using cStock routinely to send receipt messages upon collecting their products. Most (96% in EM and 87% in EPT) of the HF staff interviewed reported always using cStock to inform HSAs of orders ready for collection; 44% of HF staff in EM and 30% in EPT used it for follow–up on non– and incomplete reporting, both critical SC performance indicators, particularly for the DPATs.

FGD findings show that cStock contributed to improved accountability for managing products, as HSAs, HFs, and district staff were all responsible for and played roles in reporting and communicating stock–related issues. One positive result of this is reduced product wastage; an HSA Supervisor from the EM group explained “*There is more transparency now as drugs are not given on a friendly basis, when you find products are ready, we count with the specific HSA for accountability...*”

According to FGD participants, cStock is an important source of feedback and information. HSAs perceive monthly cStock reminders about reporting as useful, and HF staff noted that cStock alerts are beneficial in notifying them of late reporting from HSAs. An HSA in the EPT group shared “*l feel very happy whenever l am sending SMS to cStock because l receive feedback right away and because of this I am motivated*.” An HSA Supervisor from EPT adds, “*Yes, it’s very useful because communication is very fast between the HSA and the In–Charge, whenever they want emergency order they receive feedback right away from the health facility*.” cStock provides useful information for monitoring SC performance. HF staff reported that cStock enables supervisors to monitor HSA performance and supervisors’ own performance through self–assessment. In the EM group, a HSA Supervisor offered “…*we look at their reporting rate, the time they sent their report, the completeness of the report and supervisor notes that, on cStock helps us to monitor performance as the information is easily available*.”

FGD participants felt that cStock increases the overall efficiency of the community supply chain system. HSAs reported that cStock has resulted in a marked reduction in the significant effort, time, and/or money they had previously spent in collecting products from the HF. One HSA in the EM group shared, “*as my friends have said, transport costs have been reduced significantly and that is good for us*.” HF staff reported being able to manage inflow of HSAs to the HF to collect products by picking and packing in advance as well as sending notifications to HSAs that their order is ready thus serving them better. cStock also increases staff ability to monitor and communicate information on product usage. HSAs reported that cStock allows tracking product distribution and use, both at the HSA Supervisor level and higher. An HSA from the EPT group added, “*… there is transparency on cStock that your boss is able to know if the products are distributed*.”

### Acceptability of EM (added effect of DPAT)

Acceptability of the DPAT component of the EM intervention was evaluated by looking at how districts, HF staff and HSAs used information supplied by cStock for routine monitoring and managing of community SC performance, improving on their DPAT targets and rewarding well performing HSAs. All district staff in EM districts reported visiting the dashboard at least once a week to obtain data from cStock for planning, coordination, and supervision. District staff also printed cStock reports for use by HF staff to monitor HF and HSA performance. HF staff reported using cStock information obtained either through the resupply worksheets or cStock reports to discuss reporting (79%) and stock management (53%) during DPAT meetings. Seventy–eight percent of the HSAs said their supervisors referred to the performance targets such as reporting during the DPAT meetings and 80% knew they would receive rewards if they performed well in stock management using cStock. **Table 3** presents summary of acceptability findings for DPATs from the follow up survey.

One aspect that made EM acceptable to users was the DPAT role in monitoring SC performance. HSAs reported that DPAT meetings were useful for discussing SC issues, such as HSA performance, reporting rates, and performance targets. One example from an HSA in the EM group is “*sometimes it happens that you receive less products when you see more cases, we discuss how best to cater for these cases*.” HSA Supervisors reported discussing similar topics, such as HSA performance, supply management, stock outs, reporting rates, and recognition of good performance among HSAs. And HSAs described being motivated by the performance monitoring, positive feedback, and performance targets used as part of the DPAT design. Another HSA in the EM group stated, “*for us we share ideas during the meeting, as a single person you cannot build a house, we are motivated*.” HF staff also cited DPAT meetings as useful in providing a forum for reviewing SC issues and performance.

HSAs and HF staff said that EM helped link people and activities across the supply chain, aligning the flow of information and clarity of processes. Challenges to DPATs included HSA attendance, HSA hunger when no food or refreshments were offered, lack of follow up on issues discussed at meetings, lack of needed materials, and poor coordination/communication by HF In–Charge.

In terms of comparison with the EPT group, performance on most acceptability indicators in **Table 3** was slightly higher in the EM group. For example, district staff in the EM group used cStock more than their EPT counterparts, with 2 of 3 (67%) EM district staff using cStock to target health facilities for supervision compared to 0 of 3 in EPT.

### Effectiveness: supply chain performance of EM vs EPT groups

Findings on effectiveness are presented as a comparison of supply chain performance between the EM and EPT groups according to these four indicators: reporting, complete reporting, lead time, and stock out rates over time, using data from cStock dashboard reports.

### Reporting rates

**Figure 3:**
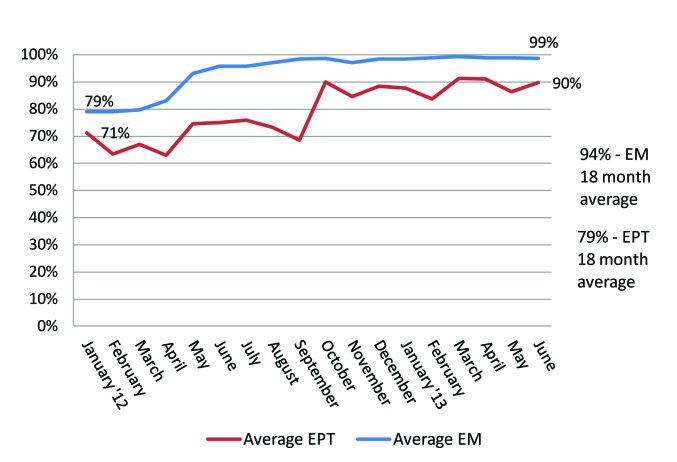
Mean reporting rates to cStock by HSAs, on all commodities in EM (Enhanced Management, n = 393) and EPT (Efficient Product Transport, n = 253) districts, January 2012 – June 2013.

Analysis of cStock results for January 2012 – June 2013 showed higher mean reporting rates in EM (94%) compared to the EPT (79%) group. There was a slow increase in time for both groups, and greater variation seen within the EPT group than EM. This study found that the EM group had significantly higher reporting rates (94.0 ± 10.7%) compared to the EPT group (79.1 ± 11.0%), *t*_(106)_ = 6.9766, *P* < 0.001 (**Figure 3**).

### Complete reporting

**Figure 4:**
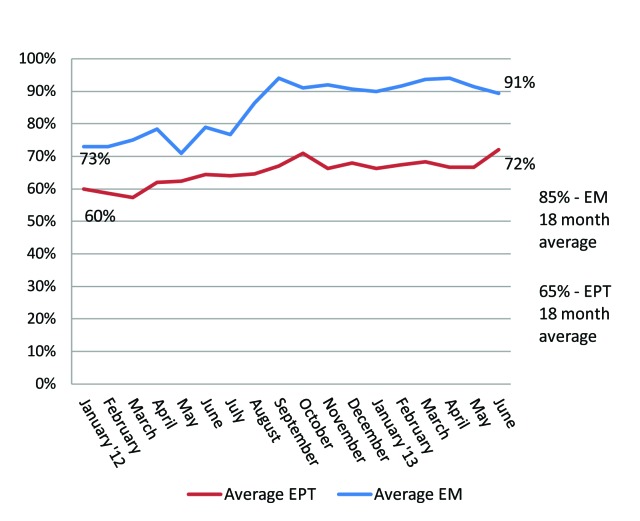
Mean reporting completeness by HSAs (health surveillance assistants), on all commodities, in EM (Enhanced Management, n = 393) and EPT (Efficient Product Transport, n = 253) districts, January 2012 – June 2013. Asterisk – findings from a two sample t–test with equal variances suggest that the differences in means between the EM and EPT districts are statistically significant (*P* < 0.001).

Completeness in reporting measures the extent to which HSAs send in stock on hand messages to cStock for all the products they manage. Mean completeness rates were found to be higher among EM (85%) than EPT districts (65%). **Figure 4** shows the trends in complete reporting between the two groups over 18 months. This study found statistically significantly higher completeness rates (85.0 ± 9.7%) in the EM group compared to the EPT group (65.2 ± 11.1%), *t*_(106)_ = 9.8953, *P* < 0.001.

**Figure 5:**
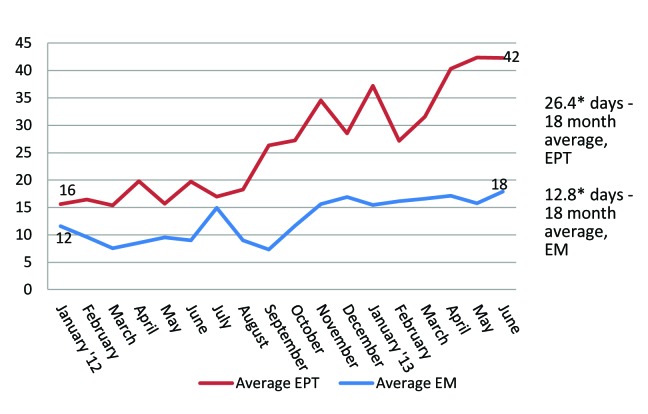
Lead time for HSA (health surveillance assistant) drug resupply calculated by cStock, in number of days, in EM (Enhanced Management, n = 393) and EPT (Efficient Product Transport, n = 253) districts, January 2012 – June 2013. Asterisk – findings from a two sample t–test with equal variances suggest that the differences in means between the EM and EPT districts are statistically significant (*P* < 0.001).

**Lead times.** On average, lead times, a measure of responsiveness in the supply chain, were significantly (*P* < 0.05) lower/better in EM compared to EPT districts with HFs in the EM group taking on average 12.8 days to fulfill an HSA order compared to 26 days in the EPT group. The difference in the two groups was small (four percentage points) in Jan 2012, increasing (to 24 percentage points) in June 2013. This study found statistically significantly lower lead times (12.8 ± 6.2) in the EM group compared to the EPT group (26.4 ± 11.3), *t*_(106)_ = –7.75, *P* < 0.001 (**Figure 5**).

### Supply reliability

**Figure 6:**
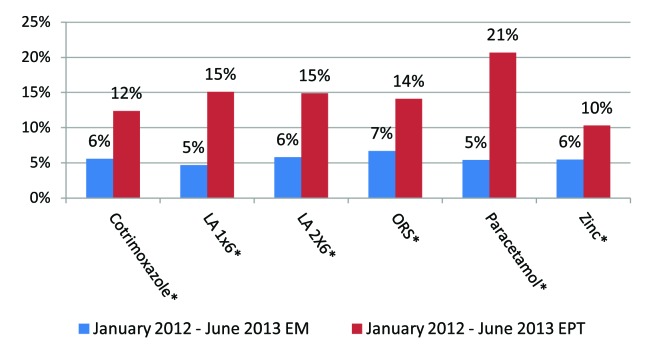
Mean percentage stockout rate over 18 months, by product, for EM (Enhanced Management) vs EPT (Efficient Product Transport) districts, (January 2012 – June 2013). Asterisk – *P* < 0.001.

Supply reliability can be defined as consistent availability of health products required in the community to treat sick children [10]. Reliability results are presented here as a measure of mean percent HSA stock out rates by product. cStock data analyzed for six iCCM products over an 18-month period ranged between 5–7% stocked out for all products in the EM group and between 10–21% in the EPT group. These differences were statistically significant at the *P* < 0.001 level for all products. The difference in stockout rates was largest in the case of Paracetamol with mean stockout rates of 5% in the EM group districts and 21% in the EPT group districts (**Figure 6**).

## DISCUSSION

Our results in both study groups demonstrate that cStock was considered feasible and acceptable to users at all levels in Malawi, and that based on comparison with the EPT group, the DPAT component of the EM group is an essential pairing with cStock to achieve the best possible SC performance and supply reliability among HSAs. Findings on the feasibility of cStock demonstrate that over 70% of community and facility based providers in both study arms were able to send and receive messages without any challenges. Among the few providers experiencing difficulty, challenges were noted as poor network penetration, particularly in mountainous districts, and limitations in the availability of phone chargers. At district level, dashboards used to synthesize data were similarly reported to be easy to use and accessible. The feasibility results also point to the importance of design considerations in development of a mHealth system. cStock was designed as a simple and user friendly system that aligned with the country’s existing health information reporting systems and staff capacity to perform SC tasks which facilitated adoption and continued uptake by the users.

The acceptability of cStock—measured through routine use and perceived benefits – increased over time, and over an 18–month period the mean use rates for reporting stock levels were 94% among EM districts and 79% in EPT. cStock acceptability levels were high in both groups, as the system was found to be beneficial in supporting essential SC tasks such as resupplying HSAs. This made the work of HF staff less burdensome and more efficient as it helped prevent unnecessary distribution of drugs through automated calculation of resupply quantities and improved accountability and transparency in stock management. Usage rate among HSAs, also an indication of feasibility and acceptability of the system, were high from inception (compared to BL data from paper reports) and sustained as shown by the data extracted from cStock over 18 months. The study also found that following training, HSAs readily adopted and routinely used cStock as an alternative method for ordering products on their own volition without institutional strength ordering them to do so. This rapid uptake of cStock occurred even with the existence of a government–approved paper system that served the same function and can be attributed to the immediate perceived benefits that included a communication and feedback loop related to product requests. The high cStock reporting rates sustained over time despite the presence of an alternative mechanism for sending reports are a further indication of user preference and acceptability of the system. Additionally, empirical evidence from the cStock dashboard shows that HSAs continued using cStock after the pilot at nearly the same or similar usage rates at the follow up assessment.

The difference noted in reporting rates across the two study groups demonstrates the added benefit of DPATs – teams created in EM districts to enhance data utilization and reward HSAs for good performance of SC tasks. While feasibility and acceptability were equally high in both groups, the enhanced effectiveness shown by improvements in timely reporting, and reductions in lead time and stock outs in the EM group can be also be attributed to the DPAT component, which laid emphasis on the use of data for decision making to drive continuous improvements and recognize good performance. DPATs facilitated much of the continued use of data by HF staff in EM districts for monitoring and management, and had a clear and strong association with improvements in all measures of effectiveness–– complete reporting rates were on average 20% higher in the EM group, lead times/responsiveness to orders were half as long and mean stock out rates half as high in EM compared to EPT districts. Compared to the paper based reporting system assessed at baseline, where districts only had access to HSA logistics data from 29% of HFs, now they are able to have timely access to data from over 80% of HSAs. Equally important, due to the design and acceptability of DPATs, HF and district managers use cStock data for resupply, for monitoring priority indicators such as reporting rates, and for management decision making to improve outcomes such as supply reliability. These findings show that not only is a feasible, acceptable system like cStock for supplying data beneficial, the higher performance on SC indicators in the EM group clearly points to the added value of DPATs, which, when integrated within existing health organizational systems and structures, can provide a mechanism for making data more meaningful and useful.

### Generalizability of findings

Although there is a growing evidence base for implementation of mHealth systems to solve public health problems, only a limited number of solutions aim to improve supply chain effectiveness [11]. To the best of our knowledge, our study is the first to address feasibility, acceptability and effectiveness of a mHealth solution that targets CHWs at the last mile of supply chains. Our findings on the use of mHealth to improve data visibility and use are consistent with the studies conducted by SMS for Life in Kenya and Tanzania and an SMS-based malaria reporting system supported by Rapid SMS tested in Uganda at peripheral health facilities (one step above the last mile), that demonstrated that mobile technology can improve reporting rates and lead to a reduction in stock outs if managers respond to the timely data [12–14].

The importance of exploring ways to promote routine system use and enhance acceptability is demonstrated by the SMS for Life study in Kenya and Tanzania, where financial incentives were provided for reporting stock information, resulting in higher reporting rates than in the Uganda study, where incentives were not used. The Uganda study however recognizes that better impact related to stock management was likely if district teams were fully engaged, emphasizing the importance of such reporting systems being nested within organization support structures. Similarly in Malawi, the implementation of cStock was integrated with additional components in the two intervention groups designed to enhance the overall performance of the supply chain; the findings from the EM group suggest that the team approach had an additive and significant effect in further improving SC performance for reporting rates, reporting completeness, lead times (responsiveness of the supply chain) and stock out rates (supply reliability). This finding is in agreement with the broader discussion by experts in the field of mHealth who have suggested that integration of mobile–based tools with existing health management systems and structures will be critical to national level adoption and scaling up of such programs [15]. We believe this is one of the first papers that has tested the hypothesis that while a viable mobile–based tool like cStock is important, the integration of information flow systems with management structures such as DPAT is critical to the tools’ adoption and ability to achieve scale and long term effectiveness.

### Limitations

One of the greatest challenges in implementation and evaluation of cStock was related to the complex supply chain and product management environment in Malawi. Due to the economic crisis in the country iCCM implementing partners were provided funding by their donors to supplement the national supply of iCCM medicines and distribute products directly to HSAs. These parallel donor–funded distribution systems bypassed the government–funded public supply chain and therefore masked true product availability throughout the supply chain. Thus attribution of improvements in product availability as a result of the EM and EPT interventions was not possible to determine and supply reliability was used as an indicator instead. Additionally, the scope of this research was limited to analysis of effectiveness of cStock and DPATs (EM) in terms of supply chain outcomes, and did not delve into the additional cost and cost saving related to either component of this integrated approach.

## CONCLUSIONS

Our findings suggest that cStock is a feasible, acceptable and effective tool for improving community health supply chain management. The integration with DPATs to promote the timely and appropriate use of system generated data to strengthen community health supply chain outcomes should be considered as a context–specific adaption for countries interested in adopting cStock. We hypothesize that establishing multilevel teams serves to connect HSAs with decision makers at higher levels of the health system, align objectives, clarify roles in the supply chain and promote trust and collaboration, thereby laying the foundation for country ownership and scalability of a cStock–like system. Continued research around the factors contributing to scalability and sustainability, as well as cost–effectiveness of such interventions, will be critical to strengthen the evidence base in this area.
